# Evaluation of the Characteristics Associated With Methamphetamine Use in Patients With Heroin Use Disorder

**DOI:** 10.31083/AP49341

**Published:** 2025-12-22

**Authors:** Zeki Vatansever, İzgi Alnıak, Tonguc D Berkol

**Affiliations:** ^1^Department of Psychiatry, Tekirdag State Hospital, 59000 Tekirdag, Turkiye; ^2^Department of Psychiatry, Bakirkoy Research and Training Hospital for Psychiatry, Neurology and Neurosurgery, 34147 Istanbul, Turkiye

**Keywords:** heroin, methamphetamine, substance-related disorders, depression, suicidal ideation, Brief Psychiatric Rating Scale, cross-sectional study, exploratory behavior, patient compliance

## Abstract

**Objective:**

This study compared addiction severity, psychotic symptoms, suicide risk, and craving in patients with heroin use disorder, with and without methamphetamine use. We also investigated the reasons for methamphetamine use in these patients, and assessed 3-month clinical follow-up and treatment compliance.

**Methods:**

This cross-sectional study included 166 inpatients diagnosed with heroin use disorder (DSM-5). Patients were divided into two groups: heroin use only (H), and heroin use + methamphetamine use (H+M). Clinical assessments included the Addiction Profile Index-Clinical Form (API-C), Brief Psychiatric Rating Scale (BPRS), and Suicide Probability Scale (SPS). Statistical analyses were conducted with Statistical Package for the Social Sciences (SPSS) and included descriptive statistics, Kolmogorov-Smirnov test, Chi-square test, Mann-Whitney U test, and logistic regression. Three-month follow-up results and treatment compliance were compared between the two groups.

**Results:**

The H and H+M groups included 80 and 86 participants, respectively. The H+M group had higher BPRS total scores, API-C subscale scores (craving, risky behaviors, excitement-seeking, impulsiveness, depression), addiction severity, additional substance use, anxiety, depressive symptoms, suicidal ideation, and 3-month lapse rate. Craving and excitement-seeking were independent predictors of methamphetamine use.

**Conclusion:**

The H+M group showed more severe addiction, novelty-seeking personal characteristics, and suicidal ideation compared to the H group. Craving scores were higher in the H+M group and should not be overlooked, along with a greater risk of early lapse. Our study found that craving, risky behaviors, depressive and psychotic symptoms, and suicidal thoughts are the most critical issues to be addressed in the treatment and follow-up of the H+M patient group.

## Main Points

1. Co-use of methamphetamine is common among heroin users, with 51.8% also using this substance.

2. Patients who consume both heroin and methamphetamine manifest more severe addiction profiles compared to those who use heroin only.

3. The use of methamphetamine was largely predicted by the craving for excitement-seeking behavior, indicating the need for targeted interventions.

4. A significant correlation between methamphetamine use and a surge in suicidal ideation was observed over a 3-month follow-up period, underscoring the significance of tailored interventions and close monitoring.

## 1. Introduction

Opioids, including natural, synthetic, and semi-synthetic derivatives, are among the oldest known psychoactive substances. They are used both medically and illicitly, with their consumption rapidly leading to addiction. Heroin addiction is the most well-known form of illicit opioid dependence [1]. Polysubstance use is common in heroin use disorder [2], with concurrent methamphetamine use rising significantly in recent years [3, 4]. In the USA, the combined use of opioids and methamphetamine is considered to be a “twin epidemic” [5]. Following various regulatory measures, the surge in prescription opioid abuse has led to an increase in the use of other substances [3, 5].

**Fig. 1.**
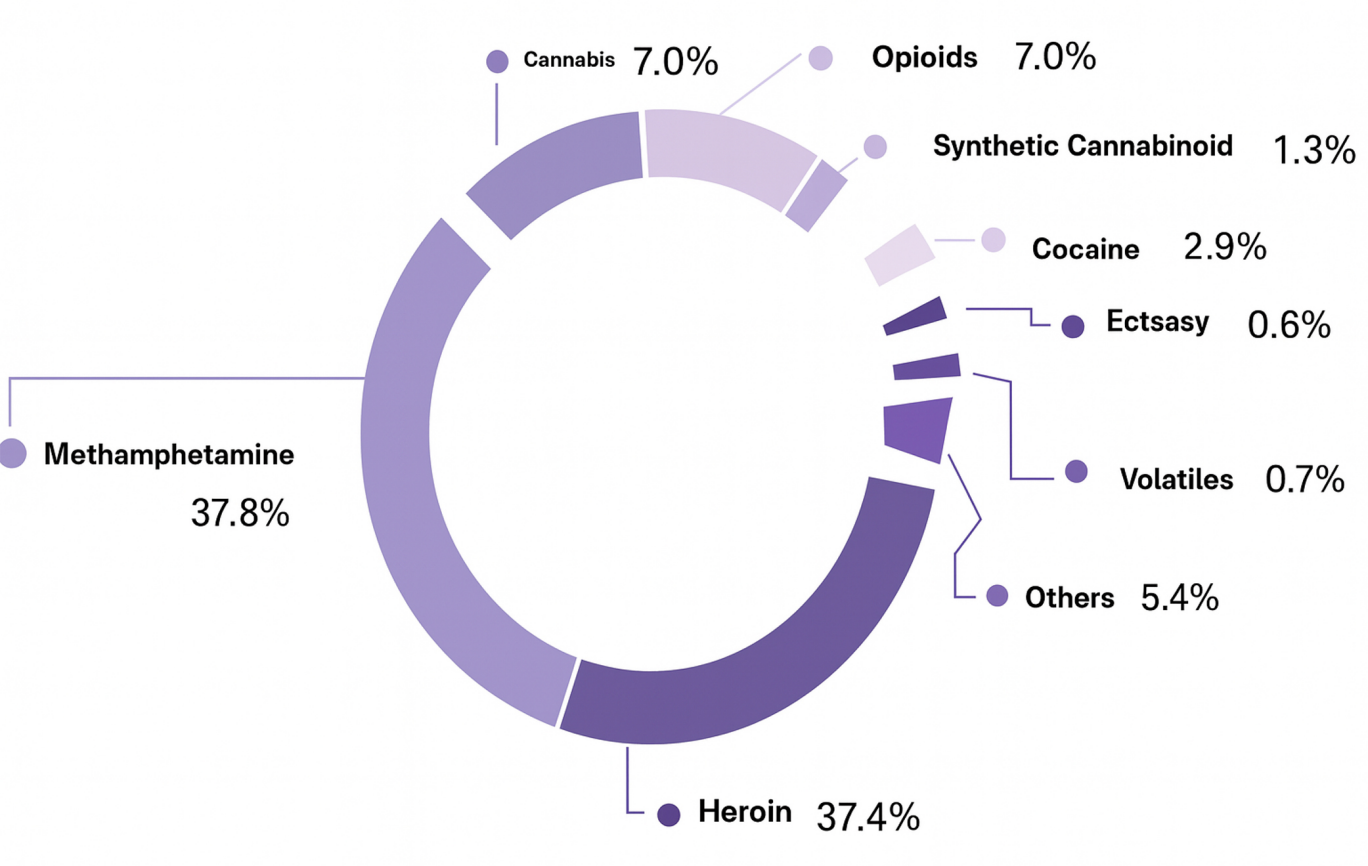
**According to the 2023 Turkiye Drug Report, the distribution of substance users according to substance types**.

Between 2010 and 2020, the seizure of methamphetamine in the EU increased by 477% [6]. In the USA, the use of methamphetamine among heroin users increased from 9% in 2015 to 30.2% in 2017 [7]. Significant methamphetamine use has also been reported among patients receiving opioid agonist treatment [8, 9, 10]. While no prevalence information exists for Turkiye, both European [11] and Turkish Drug Reports highlight record-breaking methamphetamine seizures, making it the second most commonly used substance among individuals seeking treatment [12, 13] (Fig. 1). As reported by Turkish Monitoring Centre for Drugs and Drug Addiction (TUBİM) [13] and other clinical study, methamphetamine use is widespread, particularly among heroin users [14]. A survey of Turkiye’s probation population found that methamphetamine was the second most commonly used drug (24.4%), with 51.9% of heroin users also using methamphetamine [14]. Additionally, 9.5% of lifetime heroin users currently use methamphetamine, whereas 3% of lifetime methamphetamine users currently use heroin [15].

Several studies have shown that combining opioids with methamphetamine leads to more psychiatric and medical complications than opioid use alone, highlighting the need for specialized treatment [2, 16, 17, 18]. These complications include psychotic disorders [19], risky substance use (e.g., intravenous (IV) drug use) [3, 20, 21], risky sexual behaviors [16], infectious diseases (hepatitis, human immunodeficiency virus (HIV)) [4, 21], overdose [22], and an increased risk of death [4, 7].

Studies have also indicated that combined heroin and methamphetamine use leads to a worse clinical course, lower rates of treatment-seeking [17, 23] and compliance [23, 24], higher relapse rates [25], and increased criminal issues [16, 26]. Among untreated opioid users, those using amphetamine-type stimulants differ in socio-demographic and health-related factors during emergency visits [27]. Heroin users who consume methamphetamine tend to be younger, have lower socio-economic status, and face more criminal, medical, and psychiatric issues [5, 27]. Additionally, patients with co-occurring heroin, methamphetamine, and other substance use disorders show increased rates of depression, social phobia, and anxiety, thereby altering their prognosis [28].

Multiple factors contribute to the widespread use of methamphetamine among opioid users [3]. These include reduced opioid availability, prescription opioid restrictions [3, 5], the synergistic “high” effect obtained with methamphetamine, the ability of methamphetamine to counter opioid sedation and withdrawal symptoms [29, 30], and its lower cost and accessibility compared to heroin. Some users perceive methamphetamine as a safer alternative because of the risk of opioid overdose [16, 31].

The timing of combined heroin and methamphetamine use significantly increases the risk of overdose and death [32]. Ellis *et al*. [5] found that 80% of users took both substances on the same day, with 38.9% taking them simultaneously, 9.4% immediately before and after, and 31.5% at different times. In contrast to the misconception that methamphetamine prevents heroin overdose, its combination with opioids actually increases the risk of overdose and death [32]. In Turkiye, 62.2% of drug-related deaths in 2022 involved multiple substances, with methamphetamine present in 52.3% of polysubstance-related deaths [13]. The combined use of these substances has severe physiological effects, including increased respiratory depression, cardiovascular strain, and riskier substance use behavior, such as high-dose IV injection [16, 29, 33].

The aims of this study were to determine the prevalence of methamphetamine use among patients seeking treatment for heroin use disorder, to compare the socio-demographic and clinical characteristics of heroin-only and combined-use groups, and to assess the impact of combined use on treatment outcomes. This is one of the first research studies in Turkiye to explore the clinical features, motivations, and relapse outcomes linked to methamphetamine use in patients with heroin use disorder. It therefore provides valuable insights into increasing current interventions in areas with similar trends.

## 2. Methods and Materials

This cross-sectional study included 166 inpatients diagnosed with Heroin Use Disorder as per the DSM-5 criteria, with or without methamphetamine use. Patients were diagnosed at the Adult Detoxification Center (AMATEM), Prof. Dr. Mazhar Osman Mental Health and Nervous Diseases Training and Research Hospital, University of Health Sciences, Istanbul, Bakırköy. This study was not a clinical trial. The participants gave their consent to participate after receiving verbal and written information. If available, a researcher-prepared data form was used to assess socio-demographic and clinical characteristics, supplemented by medical records and information from relatives. Detailed clinical information included age at substance initiation, duration, frequency, amount, route, last use, and treatment history. All participants underwent standard urine toxicology tests to confirm substance use. The study also leveraged a structured item on the clinical data form to assess the participant’s casual sexual partner relationships. The participants were expected to give affirmative responses if they had sexual intercourse with someone they were not committed to over the past year. The researchers coded the responses and validated them with clinical interview data.

## 3. Measurements

The study employed three instruments to test various factors. The first was the Addiction Profile Index-Clinical Form (API-C). Ögel *et al*. [34] initially developed the Addiction Profile Index (API) in 2012. This self-reported questionnaire comprises 37 items that assess the severity and characteristics of substance use with subscales listed under Table 1 [35]. In 2015, the authors developed the API clinical version (API-C) to collect participant information regarding substance use patterns and mental health dimensions. API-C evaluates six aspects that persist and coexist with addiction: impulsivity, novelty-seeking behavior, lack of safe conduct, anger control failure, anxiety, and depression [35]. For the 58 items in API-C, scores above 14 indicate a high level of addiction, scores between 12 and 14 show moderate addiction, while scores below 12 indicate low addiction. Reliability and validity of the API-C is examined by the researchers [35].

**Table 1.** **Comparison of scale scores of groups**.

	H	H+M	Test stat.	*p*
^2^BPRS total	6.50 (4.00)	9.00 (6.50)	2.932	0.003
API				
^1^CSU subscale	2.37 ± 1.34	2.79 ± 1.42	–1.962	0.051
^1^Diagnostic subscale	17.16 ± 4.43	18.41 ± 4.18	–1.872	0.063
^1^Impact on life subscale	30.16 ± 6.03	31.56 ± 6.05	–1.487	0.139
^1^Craving subscale	9.51 ± 3.73	11.40 ± 3.36	–3.420	0.001
^1^Motivation subscale	11.11 ± 1.84	10.83 ± 1.87	0.996	0.321
^1^API total	13.14 ± 2.37	14.08 ± 2.32	–2.566	0.011
^1^Lack of anger control	2.88 ± 1.66	2.90 ± 1.53	–0.082	0.934
^1^Lack of safe behavior	4.05 ± 2.21	5.40 ± 2.32	–3.817	<0.001
^1^Excitement–seeking behavior	1.88 ± 1.41	2.71 ± 1.64	–3.508	0.001
^2^Impulsiveness	3.00 (2.00)	3.00 (1.25)	2.116	0.034
^1^Depression	3.68 ± 1.78	4.58 ± 2.09	–2.990	0.003
^1^Anxiety	2.31 ± 1.39	2.72 ± 1.65	–1.717	0.088
SPS				
^1^Hopelessness subscale	27.83 ± 6.11	27.94 ± 5.33	–0.132	0.895
^1^Suicidal ideation subscale	12.56 ± 5.30	14.4 ± 4.80	2.147	0.033
^1^Negative self–evaluation subscale	21.98 ± 4.57	22.81 ± 5.40	–1.077	0.283
^1^Hostility subscale	12.34 ± 3.73	13.35 ± 3.67	–1.759	0.080
^1^SPS total	74.70 ± 12.35	78.35 ± 12.97	–1.853	0.066

H: Group with heroin use disorder but no methamphetamine use; H+M: Group with heroin use disorder and methamphetamine use; API, Addiction Profile Index; BPRS, Brief Psychiatric Rating Scale; SPS, Suicide Probability Scale. ^1^Student *t*-test, ^2^Mann Whitney U, Median (Interquartile Range) test, Mean + Standard deviation.

The second instrument employed in this study was the Brief Psychiatric Rating Scale (BPRS), initially created to evaluate the psychopathology of multiple psychiatric disorders. BPRS assesses the severity of psychiatric symptoms, particularly those related to psychosis. It has from 16 to 24 items that are rated on a 7-point Likert scale ranging from “1” (not present) to “7” (extremely severe) [36]. Participants score at least 18 points, with a higher score indicating more severe symptomatology. The BPRS demonstrated a Cronbach’s alpha of 0.87 [36].

The third measurement instrument was the Suicide Probability Scale (SPS). SPS is a self-reported scale with 36 items assessed on a 4-point Likert scale that evaluates the suicide risk of participants [37]. The dimensions encompass hostility, negative self-assessment, suicidal ideation, and hopelessness. The total score can range between 36 and 144, with higher scores reflecting a higher suicide probability [37]. Researchers have previously examined the reliability and validity of the SPS scale in a Turkish population [37]. The three instruments described above were administered to all participants, with the heroin-only group (H) compared to the combined heroin and methamphetamine use group (H+M). Patients were followed up for three months to assess differences in disease progression and treatment compliance between the two groups.

*Inclusion Criteria*:

1. Diagnosed with heroin use disorder according to DSM-5;

2. 18–65 years of age;

3. Literate;

4. Agreed to participate in the study and gave written consent.

*Exclusion Criteria*:

1. Less than 18-years old, or over 65-years old;

2. Refused to participate in the study;

3. Psychiatric illness due to mental retardation, dementia, or general medical condition;

4. Substance intoxication.

Initially, 182 patients were assessed for eligibility. Following application of the inclusion/exclusion criteria, 16 participants were excluded for the following reasons: 7 refused to participate in the study, 4 did not meet the age requirement, 3 had underlying severe psychiatric illness, and two were intoxicated with other substances. The final study cohort therefore consisted of 166 participants. Some variables had missing data due to incomplete patient records or unavailability of information. These cases were excluded from the corresponding statistical analyses.

The power analysis was conducted using Python 3.10 with the statsmodels 0.14 package (Python Software Foundation, Wilmington, DE, USA). A two-sample *t*-test with an effect size of 0.3 determined that 175 samples were needed for two groups based on α = 0.05 and an expected power of 0.80. This was based on the expected medium effect size (Cohen’s *d* = 0.3) for the difference in total API score (the primary outcome measure) between two independent groups, H and H+M.

Statistical analyses were performed using the Statistical Package for Social Sciences (SPSS) 29.0.2.0(20) (IBM Corp., Armonk, NY, USA) for macOS. Descriptive statistics (mean, ratio, and standard deviation) were calculated. The normality of continuous variables was assessed using the Kolmogorov-Smirnov test, visual inspection of histograms and Quantile–Quantile (Q-Q) plots, and evaluation of skewness and kurtosis values (values within ±1 were considered acceptable for normality), while categorical variables were compared using the chi-square test. The Mann-Whitney U test was used for pairwise comparisons of non-parametric data where the Student *t*-test was used for parametric data, and Pearson and Spearman’s rho correlation analyses were used to examine relationships between variables. Univariate regression analysis was conducted on clinical, sociodemographic, and index/scale data to predict methamphetamine use. A multivariable logistic regression model was constructed by considering the variables found to be significant in the univariate analyses, along with certain clinical variables emphasized in the literature. Statistical significance was set at *p* < 0.05.

## 4. Results

### 4.1 Socio-Demographic Data

The study included 166 patients with heroin use disorder, including 158 males (95.2%) and eight females (4.8%). Of these, 80 patients (48.2%) had heroin use disorder with no methamphetamine use (H group), while 86 (51.8%) also used methamphetamine (H+M group). The mean age was 32.23 ± 7.12 years in the H group and 31.34 ± 6.14 years in the H+M group, with no significant difference (*p* = 0.39).

The mean years of education were 8.63 ± 2.89 for the H group and 8.30 ± 2.85 for the H+M group (*p* = 0.47). Marital status was similar between the two groups (*p* = 0.506). In the H group, 60.0% (n = 48) were single, 31.3% (n = 25) were married, and 8.7% (n = 7) were divorced/widowed, while in the H+M group, 66.3% (n = 57) were single, 23.3% (n = 20) were married, and 10.4% (n = 9) were divorced/widowed.

Employment status also showed no significant difference between the two groups (*p* = 0.379). In the H group, 28.7% (n = 23) had regular jobs, 15.0% (n = 12) worked irregularly, and 56.3% (n = 45) were unemployed, while in the H+M group, 30.2% (n = 26) had regular jobs, 8.1% (n = 7) worked irregularly, and 61.6% (n = 53) were unemployed.

Regarding living arrangements, the majority of participants in both groups were living with their families, with 91.3% (n = 73) in the H group and 89.5% (n = 77) in the H+M group, while a smaller proportion lived alone or with others (8.7% [n = 7] in the H group and 0.5% [n = 9] in the H+M group). This difference was not statistically significant (*p* = 0.708).

### 4.2 Clinical Data

Table 2 shows the clinical data for the study cohort. Patients in the H+M group began substance use at a significantly earlier age than the H group (*p* = 0.022). No significant differences were found between the two groups for treatment with buprenorphine/naloxone and naltrexone, nor for the remission period with these treatments. No statistically significant differences were found between the groups for the development of substance-induced psychosis (SIP), or for related hospitalization history (*p* = 0.186 and *p* = 0.463, respectively). Moreover, no statistically significant differences were found in terms of emergency room visits, sepsis, history of wound infection, casual sexual partner relationship, relationship with two or more partners, history of sexually transmitted diseases, hepatitis B virus (HBV)/hepatitis C virus (HCV)/HIV diagnoses, family history of psychiatric diseases, or criminal history of probation and prison.

**Table 2.** **Comparison of clinical data by groups**.

		H	H+M	Test stat.	*p*
^1^ Age of starting substance use		17.00 (4.00)	16.00 (3.00)	2733.000	0.022
^1^ Duration of remission with buprenorphine/naloxone		7.00 (15.00)	5.00 (17.25)	3353.500	0.255
^1^ Duration of remission with naltrexone (months)		6.00 (9.00)	8.00 (9.00)	648.500	0.830
^4^ Duration of heroin use (years)		10.51 ± 5.02	10.59 ± 5.50	0.092	0.927
^2^ Active Intravenous Use of Heroin					
	NO	58 (72.5%)	55 (48.7%)	1.393	0.238
	YES	22 (27.5%)	31 (64.0%)		
^2^ Heroin use by shared syringes					
	NO	16 (64.0%)	13 (38.2%)	3.827	0.050
	YES	9 (36.0%)	21 (61.8%)		
^3^ History of treatment with buprenorphine/naloxone					
	NO	5 (6.3%)	4 (4.7%)	-	0.740
	YES	75 (93.8%)	82 (95.3%)		
^2^ History of treatment with naltrexone					
	NO	45 (56.3%)	50 (58.1%)	0.060	0.806
	YES	35 (43.8%)	36 (41.9%)		
^2^ SIP history					
	NO	71 (88.8%)	70 (81.4%)	1.753	0.186
	YES	9 (11.3%)	16 (18.6%)		
^2^ Hospitalization history with SIP					
	NO	71 (88.8%)	73 (84.9%)	0.539	0.463
	YES	9 (11.3%)	13 (15.1%)		
^2^ Emergency room visit					
	NO	59 (73.8%)	68 (79.1%)	0.653	0.419
	YES	21 (26.3%)	18 (20.9%)		
^2^ Sepsis wound infection					
	NO	75 (94.9%)	74 (86.0%)	3.716	0.054
	YES	4 (5.1%)	12 (14.0%)		
^2^ Random partner relationship					
	NO	49 (61.3%)	44 (51.2%)	1.712	0.191
	YES	31 (38.3%)	42 (48.8%)		
^2^ Relationships with two or more partners					
	NO	60 (75.0%)	61 (70.9%)	0.347	0.556
	YES	20 (25.0%)	25 (29.1%)		
^2^ History of sexually transmitted diseases					
	NO	76 (95.0%)	76 (88.4%)	2.358	0.125
	YES	4 (5.0%)	10 (11.6%)		
^2^ History of HBV/HCV/HIV					
	NO	68 (85.0%)	72 (83.7%)	0.051	0.821
	YES	12 (15.0%)	14 (16.3%)		
^2^ Previous diagnosis of psychiatric disease					
	NO	63 (78.8%)	60 (70.6%)	1.447	0.229
	YES	17 (21.3%)	25 (29.4%)		
^2^ Previous suicide attempts					
	NO	62 (77.5%)	66 (76.7%)	0.013	0.908
	YES	18 (22.5%)	20 (23.3%)		
^2^ History of probation					
	NO	19 (23.8%)	11 (12.8%)	3.362	0.067
	YES	61 (76.3%)	75 (87.2%)		
^2^ History of prison					
	NO	43 (53.8%)	43 (50.0%)	0.233	0.629
	YES	37 (46.3%)	43 (50.0%)		
^2^ Family history of addiction					
	NO	57 (71.3%)	58 (67.4%)	0.282	0.595
	YES	23 (28.7%)	28 (32.6%)		
^2^ Family history of psychiatric illness					
	NO	66 (82.5%)	72 (84.7%)	0.147	0.702
	YES	14 (17.5%)	13 (15.3%)		

H: Group with heroin use disorder but no methamphetamine use; H+M: Group with heroin use disorder and methamphetamine use; SIP: Substance-induced psychotic disorder; ^1^Mann Whitney U test, ^2^Chi-square test, ^3^Fischer’s Exact Test, ^4^Student *t*-test, Mean ± Standard deviation, Median (Interquartile Range), Frequency (%percentage). HBV, hepatitis B virus; HCV, hepatitis C virus; HIV, human immunodeficiency virus.

In the H+M group, 27.73% of patients stated that eliminating the sedative effect of heroin was the main reason for methamphetamine use, while 20.16% stated that it was to overcome heroin withdrawal more easily. The desire for a stronger effect was selected by 19.32%, and the need to reduce the accelerating impact of methamphetamine by 14.28%. In addition, 18.48% of subjects provided more than one reason for methamphetamine use. These findings demonstrate the diversity of motivations given by patients for combining different substances.

Among the patients of H+M group, the frequency of methamphetamine use was a median of 8.00 (Interquartile Range (IQR): 14.00) times per month, the amount of methamphetamine use was a median of 1.00 (IQR: 0.50) grams, and the duration of methamphetamine use was a median of 2.00 (IQR: 2.00) years.

The amount of heroin use was a median of 5.00 g/day (IQR: 2.88) among patients of the H group and a median of 3.00 g/day (IQR: 3.00) among patients of the H+M group; the H group had a statistically significantly higher amount of heroin use (Z = 2.23, *p* = 0.026).

The pattern of methamphetamine use was also examined. In the H+M group, 64.36% of participants stated they used heroin first and then methamphetamine, 18.39% used methamphetamine first followed by heroin, and 17.54% started using both substances simultaneously. Some subjects stated they had more than one usage pattern, thus indicating a diversity of usage habits.

No significant difference was observed between the two groups in terms of the duration of heroin use (10.51 ± 5.02 years vs. 10.59 ± 5.50 years, *p* = 0.927). Prior non-heroin substance use was significantly more prevalent in the H+M group (97.7% (n = 84) vs. 81.3% (n = 65), *p* < 0.001). No significant differences were observed between the two groups regarding the use of heroin with foil, intravenously, or nasally (*p* = 1.000, *p* = 0.238, and *p* = 0.349, respectively). The sharing of syringes was more prevalent in the H+M group (61.8% vs. 36.0%), although this reached only borderline significance (*p* = 0.050). These findings revealed differences in usage patterns between consumers of heroin alone and those consuming methamphetamine in addition to heroin.

No significant differences were found in the consumption of alcohol and cocaine between the H and H+M groups (*p* = 0.187 and *p* = 0.100, respectively). However, the H+M group had significantly higher consumption of marijuana (76.7% (n = 66) vs. 51.3% (n = 41), *p* < 0.001), synthetic cannabinoids (66.3% (n = 57) vs. 38.8% (n = 31), *p* < 0.001), ecstasy (40.7% (n = 35) vs. 16.3% (n = 13), *p* < 0.001), volatile substances (17.4% (n = 15) vs. 6.3% (n = 5), *p* = 0.027), and benzodiazepine (29.1% (n = 25) vs. 15.0% (n = 12), *p* = 0.030) compared to the H group. No significant difference was found between the two groups regarding the use of other substances (*p* = 0.095). These findings indicate that individuals in the H+M group had a higher rate of polysubstance use.

### 4.3 Comparison of Scales and Scale Scores Between the H and H+M Groups

Table 1 shows the scale scores for the two groups. A significant difference was found for the BPRS total score (*p* = 0.003).

Significant differences were also found between the two groups for the API total score (*p* = 0.011), as well as the subscales for craving (*p* = 0.001), lack of safe behavior (*p* < 0.001), excitement-seeking behavior (*p* = 0.001), impulsiveness (*p* = 0.034) and depression (*p* = 0.003). No significant differences were found for the remaining API subscales.

For the SPS scale, the only significant difference between the two groups was for the suicidal ideation subscale (*p* = 0.033).

### 4.4 Correlation Analyses

A significant positive correlation was found between the craving scores of the patients in the H group and the duration of remission with buprenorphine/naloxone treatment (*r *= 0.274, *p *= 0.018), all subscale scores and the total API scores (*r = *0.767, *p* < 0.001) except for the motivation subscale, and all subscale scores and total SPS score (*r = *0.307, *p* = 0.006), except negative self-evaluation scale. A significant positive correlation was found between all subscale scores and total scores of API (*r = *0.749, *p* < 0.001) except the API motivation subscale and the safe behavior subscale of the patients in the H+M group, and with the other subscale scores and total scores of the SPS (*r* = 0.213, *p* = 0.049) except negative self-evaluation and hopelessness.

The duration of methamphetamine use showed subscale. Data showing a significant correlation with the API craving subscale in both groups are presented in Table 3.

**Table 3.** **Correlation of Addiction Profile Index (API) craving scores with clinical data and other scale scores**.

	Groups			
	H		H+M	
	API craving		API craving	
	r	*p*	r	*p*
^2^Age of starting substance use	0.058	0.610	–0.026	0.810
^1^Duration of heroin use	–0.094	0.408	0.059	0.589
^2^Amount of heroin use	0.009	0.938	0.186	0.086
^2^Duration of remission with buprenorphine/naloxone	0.274	0.018	0.042	0.711
^2^Duration of remission with naltrexone	–0.126	0.472	–0.019	0.911
^2^BPRS total	0.179	0.113	0.117	0.283
^2^API CSU subscale	0.100	0.379	0.328	0.002
^2^API diagnostic subscale	0.399	<0.001	0.486	<0.001
^1^API impact on life subscale	0.455	<0.001	0.418	<0.001
^2^API motivation subscale	0.133	0.238	0.128	0.240
^1^API total	0.767	<0.001	0.749	<0.001
^2^API anger subscale	0.374	0.001	0.376	<0.001
^1^API safe behavior subscale	0.388	<0.001	0.201	0.063
^2^API excitement–seeking subscale	0.288	0.010	0.528	<0.001
^2^API impulsiveness subscale	0.458	<0.001	0.487	<0.001
^2^API depression subscale	0.319	0.004	0.358	0.001
^2^API anxiety subscale	0.368	0.001	0.345	0.001
^2^SPS hopelessness subscale	0.254	0.023	0.100	0.358
^2^SPS suicidal ideation subscale	0.251	0.025	0.248	0.021
^1^SPS negative self–evaluation subscale	–0.130	0.251	–0.162	0.136
^2^SPS hostility	0.326	0.003	0.424	<0.001
^2^SPS total	0.307	0.006	0.213	0.049

H: Group with heroin use disorder but no methamphetamine use; H+M: Group with heroin use disorder and methamphetamine use; CSU, Characteristics of substance use; r, correlation coefficient; ^1^Pearson correlation analysis, ^2^Spearman’s rho correlation analysis.

### 4.5 Logistic Regression Analysis

As indicated under Table 4, a multivariable logistic regression model was constructed including variables that were either statistically significant in the univariate analyses (*p* < 0.05) or highlighted in previous literature as clinically relevant cofounders. Specifically, age, duration of heroin use, API craving subscale and API excitement-seeking subscale were included based on their univariate significance or theoretical importance in polysubstance use.

**Table 4.** **Univariate and multivariable regression analysis**.

	Univariate			Multivariable		
	OR	95% CI	*p*	OR	95% CI	*p*
Age	0.811	0.638–1.030	0.086	0.973	0.912–1.039	0.413
Educational status	0.937	0.696–1.261	0.667			
Age of starting substance use	1.018	0.746–1.390	0.908			
Duration of heroin use (years)	1.336	1.004–1.778	0.047	1.037	0.955–1.126	0.390
Intravenous use of heroin	0.234	0.011–4.915	0.350			
Heroin use by shared syringe	4.903	0.790–30.436	0.088			
Family history of addiction	2.422	0.297–19.741	0.409			
BPRS total	1.023	0.850–1.232	0.807			
API craving subscale	1.785	1.105–2.883	0.018	1.114	1.008–1.231	0.034
API total	0.443	0.201–0.976	0.043			
API excitement–seeking subscale	3.221	1.191–8.716	0.021	1.293	1.014–1.649	0.038
API impulsiveness subscale	0.970	0.424–2.219	0.942			
API depression subscale	0.760	0.368–1.571	0.459			
API anxiety subscale	0.981	0.343–2.802	0.971			
Total of SPS	1.027	0.953–1.107	0.483			

According to the results of the analysis, craving level (B = 0.108, *p* = 0.034) and sensation seeking (B = 0.257, *p* = 0.038) were found to be statistically significant. In contrast, age (*p* = 0.413) and duration of heroin use (*p* = 0.390) were not found to be significant.

### 4.6 Three-Month Follow-Up Comparison of the Two Groups

Patients were evaluated at the 3-month follow-up period (early remission evaluation). The H and H+M groups showed no significant differences in the proportion of dropouts from treatment (46.5% [n = 33] vs. 53.5% [n = 38], *p* = 0.559) or outpatient treatment applications (76.3% [n = 61] vs. 69.8% [n = 60], *p* = 0.349). However, 37.0% [n = 17] of patients in the H group and 63.0% [n = 29] of patients in the H+M group had re-started the use of substances (*p* = 0.023).

## 5. Discussion

This study aimed to increase our understanding of the relationship and consequences of the co-use of opiates and stimulants, which is a growing focus of research in substance use disorder. To our knowledge, this is one of the first studies in Turkiye to systematically compare heroin-only users with polysubstance users who combine heroin with methamphetamine. The findings provide important insights into the field of addiction severity, relapse outcomes, and motivations for co-use. We utilized data from AMATEM, a nationwide addiction diagnosis and treatment center in Turkiye, to assess the prevalence, causes, clinical impact, and treatment compliance of methamphetamine use among 166 individuals with heroin use disorder. We identified significant differences in addiction severity, treatment compliance, disease characteristics, and suicidal ideation between H and H+M groups. Additionally, 3-month follow-up data provided insights into the success of early remission treatment.

The pattern of substance use varies among users [14]. In Turkiye, the widespread use of synthetic substances over the past 15 years has led to a shift in the addiction profiles seen in clinical practice [14]. Research from different regions has highlighted common issues [5, 10, 38, 39], including urgent medical and psychiatric complications, chronic health problems, and treatment challenges associated with the growing prevalence of polysubstance use. These concerns have continued to increase over time [2, 20, 21, 29].

Among the 166 patients with heroin use disorder in the present study, 86 (51.8%) also used methamphetamine, indicating that more than half of heroin users engaged in methamphetamine use. This finding is consistent with a recent clinical study in Turkiye [14]. Although there is no nationwide prevalence study, TUBİM data from 2023 show that methamphetamine use is the leading reason why patients seek treatment, thus highlighting its increasing prevalence [13].

The simultaneous use of opiates and methamphetamine has been recognized as the fourth wave of the opioid crisis in the USA. over the past decade. This has led to severe medical and psychiatric consequences, including overdose-related deaths, and posing a significant public health concern [3, 4, 5]. Similarly, methamphetamine use has become a critical issue in Turkiye. A record increase in methamphetamine seizures was first reported in 2020 [12]. This was followed by a rapid spread among substance users, leading clinicians to encounter more patients with complex medical and psychiatric conditions [13].

Medical and psychiatric histories in the present study were obtained from patient self-reports. The H and H+M groups were assessed and compared for previously diagnosed psychiatric disorders, psychiatric hospitalizations, substance-induced psychotic disorders, emergency room visits, and substance-related infectious diseases (HIV, HBV, HCV, other sexually transmitted diseases, sepsis, and IV injection site infections). However, no significant differences were found between the two groups, which was an unexpected result in view of the existing literature [3, 4, 16, 20, 21]. Notably, the quantity of heroin use and other non-methamphetamine substances was high in both groups, which likely contributed to the lack of statistical differences in medical and psychiatric complications. Additionally, many clinical parameters, such as sexually transmitted diseases and risky sexual behaviors, are based on self-reports rather than medical records or evaluations conducted during hospitalization. This could potentially lead to incomplete or inaccurate data due to patient self-presentation concerns.

Polysubstance use has become the rule rather than the exception in addiction treatment settings. This has been facilitated by the widespread use of synthetic substances, mainly because they are cheaper, more readily accessible, and because they are more addictive than traditional drugs [3, 4, 5]. Substance users sometimes try other products that are new to the drug market, without changing their preferred substances. For example, the current study found that patients did not abandon their primary substance of choice, heroin, but instead added methamphetamine to intensify the euphoric effects, navigate substance use, or manage withdrawal symptoms. The results of our study therefore support the hypothesis that existing users add new substances. Only 16.6% (n = 10) of participants used heroin only without adding other substances. A significant percentage of the respondents reported a history of using multiple substances alongside heroin, underscoring the prevalent nature of polysubstance exposure and the increasing need for personalized and adaptable treatment interventions.

Our findings also indicate that patients in the H+M group began substance use at an earlier age, potentially accelerating the development and severity of addiction. Existing research supports these findings, highlighting the increased risk of developing substance use disorder (SUD) from an early age. Previous results showed that individuals who used substances before the age of 14 were the most susceptive to developing SUD or dependence later in life [40]. The present study also found the H+M group was more likely to use other substances before heroin, indicating a pattern of escalating polysubstance use over time. This destructive pattern may be attributable to experimentation, exposure to high-risk settings that normalize polysubstance abuse, or self-medication. Such early initiation of drug use, particularly when coupled with polysubstance exposure, can result in more deeply entrenched addiction tendencies, and greater challenges in attaining sustained remission. Furthermore, addiction severity, as assessed with the API scale, was significantly greater in the H+M group than in the H group. This difference indicates a patient subgroup with more intricate and severe clinical presentations, requiring more targeted, personalized, and possibly multimodal treatment strategies.

The concepts of “substance dependence” and “substance abuse” were abandoned with the DSM-5, and substance-related disorders were evaluated under the title of “substance use disorders” [41]. An important novelty of DSM-5 was the inclusion of “craving” as a diagnostic criterion for substance use disorders [41]. In the present study, one of the subscales in the API used to evaluate addiction severity was craving. We found that craving was one of the most important contributors to the API total score. The mean craving subscale score was significantly higher in the H+M group compared to the H group. In addition, this score was one of two factors that predicted methamphetamine use in patients with heroin use disorder. Our results showed that as the craving score increased, the probability of methamphetamine use increased. There are many studies in the literature on craving in substance-using populations, and its relationship with some personality traits has been revealed [42, 43, 44]. For example, high impulsiveness was found to increase cravings, particularly in individuals who are sensitive to aversive substance-related cues [44]. Excitement- and novelty-seeking behaviors are also known to be associated with increased craving [42]. People with a prominent novelty-seeking personality are known to engage in riskier behaviors [45]. The API craving subscale score used in the current study was significantly different between the two groups and was predictive of methamphetamine use. This result was associated with some personality traits, as previously reported in the literature. For example, craving scores in both groups were positively correlated with excitement-seeking behavior and impulsiveness scores, excluding risky behaviors. However, when the two groups were compared, these personality traits were more significantly different in the H+M group than in the H group. The profile of the H+M patient group was more impulsive, excitement-seeking, and engaged in risky behaviors. Our results showed that excitement-seeking was the second most important predictive factor for methamphetamine use among heroin users, after craving.

Methamphetamine exerts psychoactive effects by inhibiting dopaminergic synapses and the destruction of monoamine oxidase [46]. Dopaminergic discharge in the limbic region plays a key role in addiction and in behaviors such as disinhibition, sexual arousal, and impulsivity [47]. Therefore, methamphetamine use is expected to increase risky behaviors in individuals with heroin use disorders. Our findings align with those in the literature highlighting the effects of methamphetamine on impulsivity and risk-taking. Although craving and excitement-seeking behaviors were found to be key predictors of methamphetamine use, the higher prevalence of risky behaviors in the H+M group cannot be interpreted solely through a cause-and-effect relationship. It remains unclear whether individuals used methamphetamine due to a predisposition for risk-taking, or if the methamphetamine use increased their risk behaviors. This could not be determined by our study design. Nevertheless, our findings provide valuable insights into the addiction profiles of methamphetamine users. Notably, the H+M group had a higher rate of use of other substances, further reinforcing their high-risk behavior pattern.

Our study investigated the motivation of patients with heroin use disorder to use methamphetamine. Subjects were asked to present reasons for using these two substances together, as they have contrasting properties, with one being a stimulant and the other a sedative. Some patients stated they began using methamphetamine to stop or reduce the amount or heroin use, while others indicated they started using it out of curiosity regarding its effects and because it was easily accessible. Some patients stated that methamphetamine was the most easily accessible drug when they could not access heroin, which was their preferred drug. With some patients, methamphetamine use almost replaced heroin use. Although heroin was the first choice in such patients, it started to become a secondary option to suppress the increased mobility, sleeplessness, and accelerating effects caused by methamphetamine use. Consequently, the motivations for initial use of methamphetamine varied between patients. In addition, it was observed that once both substances were used together, the motivation to continue using them varied. The majority of patients stated they continued to use methamphetamine because it eliminated the sedative effects of heroin. This reason is frequently mentioned in studies that evaluated the motivations for heroin and methamphetamine use [16, 29, 31]. The stimulant effects of methamphetamine may offset the sedative effects of heroin, and the resulting “balanced” feeling may lead to a perception of increased functionality. Such patients often state they use methamphetamine to “get over the sleepiness” caused by heroin, to “stay awake” for more extended periods, and to become “functional”.

The second most common motivation for methamphetamine use was to more easily overcome heroin withdrawal symptoms. In other words, methamphetamine was used as a preferred substance for heroin detoxification [29]. Previous study have reported a widespread belief that methamphetamine relieves the effects of heroin withdrawal [13]. In one study, participants explained that methamphetamine relieved them from the stress of withdrawal by reducing withdrawal symptoms and eliminating their anxiety regarding the next opioid dose [30]. A crucial result was the emphasis placed by participants on the importance of timing when using methamphetamine to relieve withdrawal symptoms [30]. The second largest group in our study consisted of patients who stated they used methamphetamine as the easiest substance to access when they could not access heroin, and that it was a “backup alternative to the substance of choice”.

The relatively high frequency of these motivations suggests that heroin is typically the initial drug of choice, with methamphetamine introduced later to fulfill a secondary need. This explains the more common pattern of heroin use first, followed by methamphetamine use. Our study found that only 33% of dual-substance users had never used the intravenous (IV) route, with the majority of IV users mixing both substances for injection.

A significant proportion of participants in our study reported multiple motivations for combined use. Some sought a more substantial euphoric effect by using both simultaneously. In contrast, others used heroin to counteract the stimulant effects of methamphetamine. Previous research on this subject suggests that genetic factors could account for some of the differences between the H and H+M groups [48]. Uludag *et al*. [48] showed that specific genetic factors were related to amphetamine use, including rs174696, rs174699, rs1544325, rs4680, rs4818, rs737866, and rs933271. Further research could lead to a deeper understanding of the role of genes in polysubstance use, thereby enabling more personalized interventions for the treatment of SUDs.

Interestingly, heroin consumption was significantly lower in the H+M group than in the H group. This may be due to the high prevalence of polysubstance use in this group, which likely reduced the overall quantity of heroin consumed.

Many studies have demonstrated the development of high rates of comorbid psychiatric disorders in individuals with substance use disorders [19, 47, 49, 50]. The lifetime prevalence of depression in heroin use disorder patients receiving treatment is 20–50% [51]. In addition, 75% of methamphetamine users have a lifetime psychiatric diagnosis, with a significant presence of depressive and psychotic disorders [47]. In addition to the depressive symptoms commonly seen in patients with heroin use disorder, psychotic symptoms and cognitive impairment have been encountered more frequently in our clinical practice in recent years. This is most likely due to synthetic substances, in addition to the preferred substance. Lopez *et al*. [16] also reported a higher prevalence of depressive and psychotic symptoms in patients using methamphetamine in addition to heroin. In our study, depressive and psychotic disorders were evaluated without distinguishing between comorbid disorders and those that developed secondary to substance use. Methamphetamine use is closely associated with psychotic symptoms and is a significant risk factor for the development of psychotic disorders [19]. The BPRS was therefore used in the present study to evaluate depressive and psychotic symptoms. The results of our study were similar to those in the literature, with the BPRS scores being significantly higher in the H+M patient group. We found positive correlations between the frequency of methamphetamine use and psychotic symptoms, especially tension, hallucinations, mannerisms and dissociation, as well as the BPRS total score. These results are noteworthy because they show that the frequency of methamphetamine use is closely related to positive psychotic symptoms.

Although positive psychotic symptoms associated with methamphetamine use were prominent in our study, depressive symptoms known to be common in patients with heroin use disorder were found to be significantly more common in the H+M group. The use of stimulants such as methamphetamine is also known to be associated with depressive symptoms, especially during withdrawal periods [47, 49]. For example, Glasner-Edwards *et al*. [52] found that depression significantly reduced functionality in methamphetamine users, and emphasized that depressive symptoms decreased after treatment for methamphetamine addiction. Our study found that depressive symptoms were more common than psychotic symptoms in the H+M group, suggesting they should not be overlooked in the treatment planning. These results also highlight the issues that must be considered for effective psychopharmacological and psychotherapeutic treatment.

Substance use disorders are among the most frequent causes of psychiatric-associated disorders [47, 53, 54]. The suicide rate of patients with opioid use disorders is reported to be 3–fold higher than the general population [55]. The association between methamphetamine use and suicide risk has also been widely reported in the literature [49, 53, 56], with one study finding that approximately one-third of deaths among methamphetamine users was due to suicide [49]. No significant difference in history of suicide attempt was found between the H and H+M groups in our study. However, the suicidal ideation subscale score assessed using the SPS was significantly higher in the H+M group than in the H group. As described earlier, the H+M group had a profile that was more depressed, impulsive, and prone to risky behaviors. Considered together with these findings, significantly higher scores for suicidal ideation in the same group should be noted. The close relationship between suicide and depressive symptoms is well-established [50]. When this close relationship occurs together with high impulsivity, it is possible that suicidal ideation may lead to suicide attempts. This relationship has been discussed in several studies [57, 58, 59]. For example, Goldston noted that patients with high impulsivity are at increased risk of suicide and substance use. The relationship between suicide and high-risk behaviors such as carrying weapons, risky sexual behaviors, and fighting has been highlighted previously [60]. Together with the results of the present study, it is clear that patients diagnosed with heroin use disorder and methamphetamine use have a different clinical profile to heroin users alone. It should be noted that suicide risk should be carefully addressed in the H+M group. Treatment should be provided for accompanying depressive and psychotic symptoms, the treatment results should be monitored, and psychotherapeutic strategies for impulsivity and risky behaviors should be meticulously planned. The consequences of cravings should also be noted at this point. The H+M group consisted of patients with higher addiction severity, consumed multiple substances together, and showed a higher level of craving. Craving is a challenging issue for all clinicians who work with the substance-using population, even during remission.

The participants in our study were followed clinically for three months, thus covering the early remission period. Some data was missing due to incomplete clinical records and failure to contact participants, while some participants withdrew from the treatment protocol without alerting the research team. Specifically, follow-up data was missing for 9 participants (5.4%), although these were retained for the baseline comparisons in appropriate circumstances. The low rate of missing data was similar between the H and H+M groups. The H+M group experienced a higher rate of lapse (i.e., restarted substance use) compared to the H group. Importantly, however, there were no significant differences between the two groups in terms of outpatient treatment applications and treatment proportion of dropouts. Even when patients started substance use again, they did not stop treatment and continued to maintain contact with the treatment team. Clinicians should take advantage of this ongoing contact to carefully evaluate the most important reasons why patients restart substance use. In addition, they should reconsider and modify the treatment according to the patient’s needs, and identify issues overlooked in the initial treatment plan. In particular, craving should be taken into account since it is more prevalent in the H+M group, and suitable therapeutic interventions for this issue should be implemented.

The results of our study also suggest that harm reduction strategies are an essential part of an integrated treatment approach for substance use disorders. Harm reduction strategies are especially important for patients with high-risk behaviors such as polysubstance use. The expansion of access to treatment for heroin-use disorders is a priority, with effective psychopharmacological treatment being one of the most essential harm reduction strategies. Opioid agonist therapy (buprenorphine/naloxone) is currently one of the cornerstones of this treatment, and its contribution to positive clinical outcomes is undeniable. Unfortunately, some of the common harm reduction strategies used in the USA and Europe are not implemented in Turkiye. These include providing access to safe consumption areas and instruments, as well as methods to prevent the transmission of infectious diseases through IV use and syringe sharing. Harm reduction strategies can help to reduce risks, given that not all patients receive complete treatment for their addiction. Additionally, patients can be made aware of the long-term effects of substance use through harm reduction strategies, and their motivation for treatment can be increased. In our opinion, making these approaches widely available in clinical practice may help to reduce the mortality and morbidity rates associated with substance use.

## 6. Limitations

Our study had several limitations. One of the most important limitations was the predominantly male study cohort (95.2%). This reflects the demographic of patients who are admitted to addiction centers in Turkiye and receive inpatient treatment for substance use [61]. Although the gender gap has narrowed in recent years [53, 56], societal stigma makes it more difficult for women to seek treatment in many countries, including Turkiye. Consequently, the number of female participants (n = 8) in our study was insufficient for gender-based comparisons. This skewed sample limits the generalizability of our findings. Despite this, methamphetamine use was notable among female heroin users, with 5 of the 8 female participants being in the H+M group. More extensive studies are needed to investigate gender differences and methamphetamine use in female heroin users.

Another limitation is that our study included only inpatients at the AMATEM Clinic Outpatients and patients who declined inpatient treatment were excluded, as were those who did not return after an initial visit, had severe substance-induced psychosis, or had a high suicide risk requiring psychiatric hospitalization. These individuals are likely to represent a different profile, meaning that our findings apply to a more defined heroin use disorder group and cannot be generalized to all heroin users.

A further limitation was that medical histories were based on self-reports and hospital records. Self-reported data may contain errors due to memory issues, under-reporting, or social desirability bias. Variability in the potency and purity of substances [55] can also influence symptoms and clinical outcomes. Moreover, concurrent substance use disorders may have affected the findings of this study.

## 7. Recommendations for Further Research

This study highlights the clinical significance of understanding methamphetamine use among individuals with heroin use disorder. The H+M group showed early initiation, significantly elevated addiction severity, impulsivity, and suicidal ideation compared to the H group. The results of our study suggest that interventions which address the underlying conditions should be tailored to the patients. Future research should therefore be aimed at:

∙ Investigating gender-specific motivations and barriers in heroin-methamphetamine co-use through larger and more diverse samples, unlike the skewed sample in the current study.

∙ Exploring the longitudinal impacts of combined stimulant-opioid use on remission and relapse.

∙ Assessing the efficacy of integrated interventions with pharmacological approaches and psychotherapy techniques for co-occurring SUDs.

## 8. Conclusions

Heroin addicts who also use methamphetamine form a distinct sub-profile characterized by more severe addiction, higher impulsivity, increased risky behaviors, and elevated suicidal ideation scores. Additionally, high craving levels increase the risk of relapse and require special attention for treatment.

This study found that methamphetamine is commonly used to counteract the sedative effects of heroin and alleviate withdrawal symptoms, emphasizing the need for effective treatment of opioid addiction. Craving, impulsivity, risky behaviors, psychotic and depressive symptoms, and suicidal ideation should be carefully monitored and managed throughout the treatment process.

## Availability of Data and Materials

The data supporting this study’s findings are available upon request from the corresponding author.
